# What about sex, race(ism), and social determinants of health in neonatal outcomes?

**DOI:** 10.3389/fped.2024.1378370

**Published:** 2024-10-01

**Authors:** Neha Chaudhary, Arushi Meharwal

**Affiliations:** ^1^ Department of Pediatrics, Tufts University School of Medicine, Boston, MA, United States; ^2^ BS Biology, College of Science, Northeastern University, Boston, MA, United States

**Keywords:** sex, race, racism, neonatal outcomes, social determinants

## Abstract

Neonatal outcomes encompass a range of outcome measures, including mortality rates, physical and mental health morbidities, and long-term neurodevelopmental statistics. These outcomes are influenced by non-modifiable factors, such as sex and race, and modifiable factors, such as social determinants of health and racism. There is a known bias toward worse outcomes for male infants in terms of preterm birth, low birth weight, and mortality, with several biological and physiological factors contributing to these sex-related differences. In relation to racial disparities, wherein race is a social construct, maternal and infant healthcare continues to lag behind for minority populations compared with the white population, despite advances in medical care. Infants born to Black women have higher infant mortality rates and lower birth weights than infants of white women. These differences can be largely attributed to social and environmental factors, rather than racial and ethnic differences. Furthermore, we emphasize the role of social determinants of health in neonatal outcomes. Factors such as economic stability, education access and quality, healthcare access and quality, the physical neighborhood environment, and the social and community context all contribute to these outcomes. Overall, this article highlights the complex interactions between sex, race(ism), and social determinants of health in neonatal outcomes. It underscores the need for a comprehensive understanding of these factors to improve maternal–neonatal care and reduce disparities in outcomes. Healthcare providers, policymakers, and communities need to work together to combat these complex issues and improve neonatal outcomes for all infants, while understanding the complex interplay between sex, racism, and/or social determinants of health.

## 1 Introduction

Neonatal outcomes is a broad term that continues to evolve depending on the duration of the interval for the follow-up of cohorts of neonatal intensive care unit (NICU) graduates and is an area of interest for the investigators. Although no clear definition exists, historically short-term outcomes are studied based on the neonatal mortality rate (NMR, defined as deaths at less than 28 days) and comorbidities, including, but not limited to, chronic lung disease, feeding problems, and retinopathy of prematurity at discharge (1). Intermediate outcomes can be assessed by the post-NMR (defined as infantile deaths at 28 days or more), infant mortality rate (IMR), and comorbidities, such as frequent hospitalizations for respiratory symptoms, a failure to thrive, and delays in attaining developmental milestones at 1 year of age (1). Long-term outcomes are generally analyzed based on longitudinal neurodevelopmental assessment data and other comorbidities defining the quality of life in survivors. NICU outcomes depend on multiple factors in the perinatal timeframe, including maternal age, parental medical health, mental health, education, socioeconomic status even before conception, challenges faced during birth hospitalization, and difficulties faced post discharge. Addressing many of these potentially modifiable risk factors can lead to improved outcomes.

**Figure 1:**
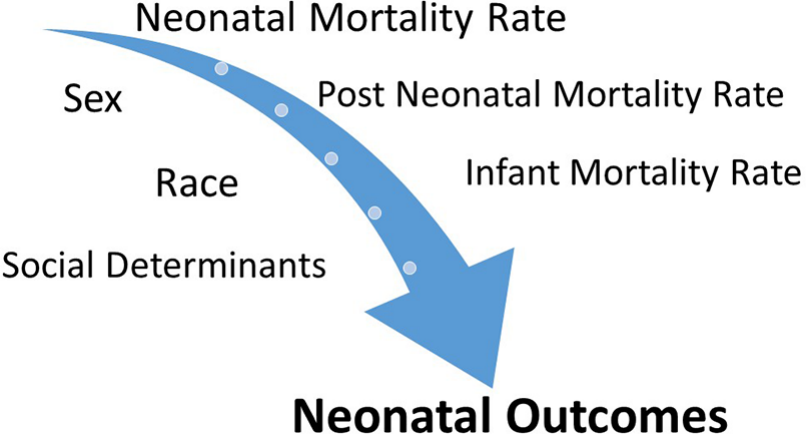
Neonatal outcomes such as NMR, post-NMR, and IMR are influenced by complex interactions between sex, race, and social determinants.

In this review article, we focus on addressing the role of non-modifiable factors (i.e., sex and race) and modifiable factors (i.e., maternal health, education, and social determinants including racism) in neonatal outcomes. There is tremendous overlap among these factors causing complex interactions and thereby influencing NICU outcomes (Figure 1). For example, birth defects, including chromosomal anomalies, are significant contributors to the IMR, which in turn could be a result of a complex mix of non-modifiable factors, such as genetics and sex, and modifiable factors, such as behavioral and environmental factors (nutritional deficiencies, maternal infections, and substance abuse). Gestational age, especially prematurity, which is another significant contributor to the IMR, determines NICU outcomes, which in turn are also influenced by sex, maternal health, and additional social factors. Many minority/underrepresented communities have experienced structural racism over generations, with the resultant financial disparities and other negative consequences attributable to inequities in the social determinants of health (SDoH) in the United States (1).

### 1.1 IMR and NMR in the United States

The US IMR in 2022 was 5.60 infant deaths per 1,000 live births and the NMR was 3.58 in 2022, a 3% rise for both from 2021 (2). The IMR, NMR, and post-NMR have steadily declined since 2005 (6.86, 4.54, and 2.32 deaths per 1,000 live births in 2005, respectively) (3). However, all of these have shown a steady increase in the last 3 years since the severe acute respiratory syndrome coronavirus disease 2019 (COVID-19) pandemic hit the world in 2019 (Table 1). This can be potentially explained by the reallocation of healthcare resources in fighting the pandemic, the potential impact of the pandemic on the pregnant population, which led to stressful pregnancies and postpartum periods, and the higher risk of preterm deliveries and physical separation of infants from their COVID-19-positive mothers early in the pandemic. Mississippi was the state with the highest IMR (9.11) in 2022 (2). The IMR significantly declined in Nevada and increased in four states—Georgia, Iowa, Missouri, and Texas (2).

**Table 1:** United States IMR, NMR, and post-NMR from 2019 to 2022.

Index (per 1,000 live births)	2019	2020	2021	2022
IMR	5.58	5.42	5.44	5.60
NMR	3.69	3.56	3.49	3.58
Post-NMR	1.89	1.86	1.95	2.02

Infant mortality data in the United States from provisional data from the 2022 period linked birth/infant death file reported by the National Center for Health Statistics (1).

These IMR and NMR indices are driven by a multitude of factors and demonstrate a strong association with ongoing neonatal and infant health disorders. Interestingly, the top five leading causes of infant mortality have remained unchanged in recent decades—congenital malformations, preterm birth/low birth weight (LBW), sudden infant death syndrome (SIDS), unintentional injury, and pregnancy complications (3). Of concern, infant mortality resulting from maternal complications of pregnancy and sepsis have increased by 9% and 14%, respectively, in the last year (2). Technological advances in medical sciences have significantly improved care provision for infants with birth defects, prematurity, and LBW, with distinct patterns of outcomes based on infant sex, with many negative associations with male sex.

### 1.2 Sex

A biological infant male is biased toward the negative outcomes of preterm birth and mortality, despite having a higher birthweight than gestational-age-matched females, and, interestingly, this has continued to be demonstrated in recent literature (4–6). These differences were further highlighted in the COVID-19 pandemic. Although there was an increase in the IMR in both sexes, the increase was disproportionate in male infants from 2021 to 2022 (Table 2). Males have a higher risk of neurological and respiratory complications than females in NICUs (7, 8). This is more apparent for extreme preterm or very LBW (VLBW) infants. Prematurity is more common in singleton male infants and male-male twins than in female-female twin/singleton gestations (8). In addition, many comorbidities, such as prematurity, necrotizing enterocolitis, intraventricular hemorrhage, and chronic lung disease, during the stay in the NICU, have a significant impact on neonatal outcomes (Table 3).

**Table 2:** Infant mortality rate per 1,000 live births based on sex and race in 2021 and 2022 in the United States.

IMR per 1,000 live births based on sex	2021	2022
Female	5.02	5.12
Male	5.83	6.06
IMR per 1,000 live births by race and Hispanic origin		
Black	10.55	10.86
Native Hawaiian/Pacific Islander	7.76	8.50
American Indian/Alaska Native	7.46	9.06
Hispanic	4.79	4.88
White	4.36	4.52
Asian	3.69	3.50

Infant mortality data in the United States from provisional data from the 2022 period linked birth/infant death file reported by the National Center for Health Statistics (2).

**Table 3:** Sex-based differences in neonatal outcomes.

Morbidity	Overall incidence rate	Male	Female	Reference
Prematurity	5.7%	6.2%	5.2%	Peelen et al. (9)
Necrotizing enterocolitis	7%	7.4%	6.4%	Jammeh et al. (10)^a^
Severe intraventricular hemorrhage	9.3%	16.1%	1.9%	Cuestas et al. (11)
Retinopathy of prematurity requiring treatment	2%	52%	45%	Hoyek et al. (12)
Severe bronchopulmonary dysplasia	n/a	59%	41%	Hammond et al. (13)
Hypoxic ischemic encephalopathy	0.1%	59%	41%	Acun et al. (14)^b^
Neonatal hypoglycemia	27%	53%	47%	Stark et al. (15)
Neonatal seizures	2.29/1,000 live births	2.38/1,000 live births	2.20/1,000 live births	Pisani et al. (16)
Cerebral palsy	2–3/1,000 live births	70%	30%	Romeo et al. (17)^c^
Neurodevelopmental impairment at 18–22 months follow-up in preterm (<28 weeks gestation at birth)		48.1%	34.1%	Hintz et al. (18)
Bayley Mental Index <70 at 18–22 month follow-up in preterm (<28 weeks gestation at birth)		41.9%	27.1%	Hintz et al. (18)

^a^ Very low birth weight.

^b^ >35 weeks.

^c^ Preterm only.

A preferential survival bias has been noted for female sex, even in fetal stages. Orzack et al. looked at male pregnancy losses and found that the ratio of male to female conceptuses was equivalent and waxed and waned during gestation (19). Specifically, in the first few weeks, there are more male fetal losses, primarily due to a higher rate of abnormalities in male embryos, followed by an increased loss of female fetuses later in the first trimester, which is followed by an increased mortality of male fetuses from mid-gestation onwards (19).

Biological sex affects innate and adaptive immune responses to self and foreign antigens (20). In addition, androgens have a role in immunosuppression, resulting in sex differences in autoimmunity and responses to infections and vaccines (21). Neuronal differentiation with the resultant sexual dimorphism in neurological disease is likely attributable to an altered sensitivity of neurons to oxidative and/or neurotransmitter stimulation (22, 23). Nuñez and McCarthy suggested that higher estradiol levels in the early embryonic phase in male development (24) and increased cerebral blood flow increased the risk of neurological disease in males (25). Furthermore, data suggest that by 34 weeks of gestation, female fetus lung maturation is approximately 1.5 weeks ahead of that in males (26, 27). Higher levels of androgen and Müllerian inhibiting substance (MIS) hamper surfactant production (28). In fact, the bias favors female survival and is more apparent in the fetal-to-neonatal transition phase. Males are also more vulnerable to respiratory distress syndrome (RDS), bronchopulmonary dysplasia (BPD), and intraventricular hemorrhage (IVH) (29) and are more likely to develop umbilical cord abnormalities, including knots and nuchal cord (30). In addition, reduced venous blood flow to fetuses with normal umbilical cords is more likely in males (31), as are obstetric complications, including gestational diabetes, placenta previa, and preeclampsia (30, 32–34). Females have a higher surge of catecholamines during stressful periods, leading to more resilient coping mechanisms. Peacock et al. found that males had a higher incidence of death and oxygen dependency. They also noted a higher incidence of poor cognitive outcomes in males that could not be explained by perinatal, neonatal, or postnatal factors (7).

In addition, preeclampsia and fetal growth restriction can be more prevalent in women carrying male babies, which means that designing individual treatment plans and encouraging pregnant women to make lifestyle changes based on the sex of their unborn babies could have lifelong health benefits for their children (35). Awareness of these negative associations of male sex with health outcomes may help guide fetal sex-based pregnancy and postnatal management.

### 1.3 Race and racism

Race has been a contentious demographic variable in research regarding its role in the true causality of disease manifestation, as it is a social construct rather than a true biological variable (36, 37). Race, if used as a surrogate measure of variations in genetic and biological structures secondary to human migration leading to the isolation of a particular population that have a common genetic descent, is considered non-modifiable (38). A population with similar genetic/biological characteristics may then additionally share similar community core beliefs, such as cultural and/or religious beliefs, further promoting similar active or passive choices in healthcare, thereby influencing neonatal outcomes. On the other hand, racism is a completely modifiable entity allowing institutions and systems to create unequal healthcare opportunities through actions and attitudes based on race. It is extremely difficult to differentiate race and racism based on demographic data alone, especially in complex social hierarchies in which social determinants play a tremendous role in shaping racism in its most tragic form. We will discuss the social determinants later in the article.

Infants of Black women had the highest IMR followed by infants of American Indian/Alaska Native non-Hispanic women in 2022 (Table 2). The post-NMR was also higher in infants of Black (4.19/1,000 live births), American Indian or Alaska Native (3.67/1,000 live births), and Native Hawaiian or Other Pacific Islander (3.36/1,000 live births) women than in infants of white (1.58/1,000 live births), Hispanic (1.52/1,000 live births), and Asian (0.90/1,000 live births) women in 2021 (2). Black women are at a higher risk of severe maternal morbidity, e.g., preeclampsia and needing ICU admission during pregnancy (39).

Are there truly different genetic/racial phenotypes for mothers and infants that influence neonatal outcomes? Table 4 notes some of the common neonatal outcomes that may be influenced by maternal race and ethnicity. The discrepancy of preterm births in different races has persisted for decades. In 2022, the rate of preterm births among African American women (14.6%) was approximately 50% higher than that among white or Hispanic women (9.4% and 10.1%, respectively) (43). Studies have shown that stress and racial discrimination are associated with the Black–white disparity in preterm births (44). Infants of Black women have a lower birth weight than infants of white women, although the growth velocity is much higher and Black infants are likely to be heavier than white infants at 18–22 months (45). Many studies have shown this difference can be attributed to social/environmental factors (45). Black and Hispanic patients are less likely to die in the NICU. However, they are more likely to be readmitted/die after being discharged (46). There is currently no definitive evidence that a single race has a genetic advantage in terms of survival in the neonatal phase. Black race is a strong predictor for intubation and resuscitation in periviable infants, likely due to cultural preferences (46). The mortality after initial discharge can be explained by a combination of environmental and social factors. In addition, there are also racial disparities in the prevalence of congenital malformations and chromosomal anomalies. American Indian women are at a higher risk of having infants with a cleft lip, Hispanic women are at a higher risk of having infants with spina bifida, and infants of Black women are more likely to die from birth defects (47). These are also largely attributable to socioeconomic disparities and racism rather than different structural genetics.

**Table 4:** Race- and ethnicity-based differences in neonatal outcomes.

Morbidity	Non-Hispanic white	Non-Hispanic black	Hispanic	Reference
Prematurity	9.4%	14.6%	10.0%	March of Dimes (40)
Necrotizing enterocolitis	Ref	aOR: 1.31 (1.25–1.39)	aOR: 1.30 (1.21–1.39)	Jammeh et al. (10)^a^
		HR: 5.02 (3.52–7.16)	HR: 2.60 (1.81–3.74)	Janevic et al. (41)^b^
Intraventricular hemorrhage	Ref	HR: 3.09 (1.79–5.34)	HR: 2.18 (1.26–3.79)	Janevic et al. (41)^b^
Retinopathy of prematurity	Ref	HR: 3.70 (2.36–5.77)	HR: 2.09 (1.31–3.36)	Janevic et al. (41)^b^
Bronchopulmonary dysplasia	Ref	HR: 5.04 (3.11–8.16)	HR: 2.22 (1.50–3.3)	Janevic et al. (41)^b^
Hypoxic ischemic encephalopathy	Ref	OR: 1.3 (1.2–1.4)	OR: 0.87 (0.8–0.9)	Acun et al. (14)^c^
Neonatal seizures	0.1%	0.02%	—	Loftin et al. (42)

aOR, adjusted odds's ratio; HR, hazard ratio.

^a^ Very low birth weight.

^b^ <32 weeks.

^c^ >35 weeks.

Based on a recent study by Horbar et al., Black, Hispanic, and Asian infants were distributed unevenly across NICUs compared with white infants. Compared with white infants, Black infants received care at lower-quality NICUs and Asian infants received care at higher-quality NICUs after accounting for the region of residence (48). The Institute of Medicine found that “bias, stereotyping, prejudice, and clinical uncertainty on the part of healthcare providers may contribute to racial and ethnic disparities in healthcare” (49). The attitude of physicians and nursing staff contributes to disparities in care, primarily because of medical staff being overwhelmed due to nurse–patient ratios and resource-limited working environments (50). Analysis of the odds of nosocomial infections and being discharged home without breastmilk in large NICUs showed that understaffing was a significant issue in hospital systems with high numbers of Black patients compared with hospital systems with low numbers of Black patients (50). Nurses in hospitals with high numbers of Black patients missed more than half of the care activities that nurses in hospitals with low numbers of Black patients undertook.

### 1.4 Social determinants of health

It is much more difficult to comprehensively tease out the contribution of SDoH to neonatal outcomes, unlike race and sex, as multiple factors come into play. They comprise all non-medical factors that contribute to NICU outcomes. Examples include stable housing, transportation, racism, income, food insecurity, single parent home, and education, which lead to stark disparities in maternal and infant healthcare that have worsened despite advances in medical care.

**Figure 2:**
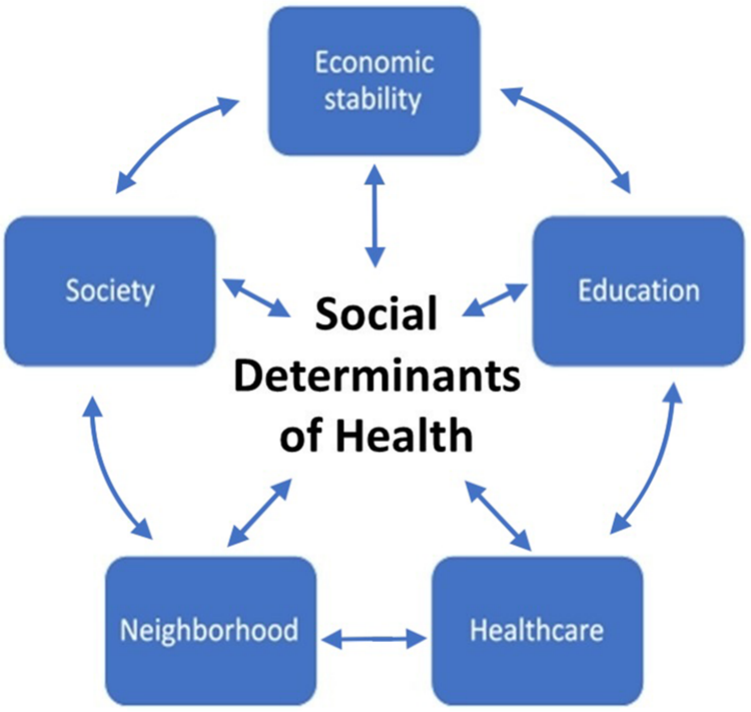
SDoH in pediatric clinical care comprises five domains that significantly overlap—economic stability, education access and quality, healthcare access and quality, the physical neighborhood environment, and the social and community context.

The Centers for Disease Control and Prevention recognizes SDoH as one of the three priority areas, in addition to health equity and health literacy, for Healthy People 2030, the fifth edition of the Healthy People initiative started in 1979 (47). Recognition of these led to the American Academy of Pediatrics (AAP) recommending universal screening for adverse SDoH in pediatric clinical care (51). SDoH entail five domains (Figure 2) that have significant overlap—economic stability, education access and quality, healthcare access and quality, the physical neighborhood environment, and the social and community context (47).

#### 1.4.1 Economic stability

In 2016, the AAP emphasized the need to focus on poverty-related risk factors in pediatric care (51). Maternal risk factors for preterm birth are socially patterned and complexly interact with medical risk factors. Low income and financial stress frequently lead to overcrowding and limit access to contraception, prenatal care, nutritious food, and personal hygiene (44). Chronic stress negatively impacts maternal health, leading to fetal growth restriction and higher preterm labor rates. The rate of preterm birth increases significantly in regions with increased poverty (44). A healthy pregnancy for a mother requires the meticulous planning of medical appointments, self-care, and care of the newborn post birth, all of which increase the financial burden on a household. Decreased physical ability due to physiological changes in the maternal body adds sick days and limits the capacity to generate income. On the other hand, financial stability leads to easier access to healthcare services. Economic stability goes hand in hand with stable employment, insurance coverage, planned debt, and medical bills (44, 51).

#### 1.4.2 Education access and quality

Women with higher education have better job opportunities and a more stable financial status. Higher education also equates to better health insurance coverage and improved resources for mental and physical health. Disrupted education, poverty, and adverse childhood experiences have been associated with teen pregnancy and are more common among Black women (39). Teen pregnancy under the age of 15 years had the highest IMR at 14.92 in 2021 (2). The provisional birth rate for teenagers aged 10–14 years was 0.2 births per 1,000 females in 2022, which was unchanged since 2015, and for teenagers aged 15–19, the rate was 13.5 births per 1,000 females, down 3% from 2021 (13.9) (43). Teen pregnancies are associated with high-risk pregnancies, prematurity, and low birth weight and continue to negatively impact maternal health and educational and job opportunities. Comprehensive sex education and an awareness of contraception has reduced teen pregnancy rates (39). Higher education can help minimize the communication barrier in health-related encounters, leading to better health literacy, efficient care, and a satisfying experience.

#### 1.4.3 Healthcare access and quality

Black, American Indian, and Alaskan Native women have higher preterm births for which they had no or late prenatal care (39). The Southeastern United States had the highest IMR in 2015 and New England and Pacific Regions had the lowest rates (52–54). Many states have not implemented the Affordable Care Act (ACA) Medicaid expansion that limits postpartum coverage for women, thereby limiting healthcare access (46). Maternity care deserts, defined as any county in the United States without a hospital or birth center offering obstetric care and without any obstetric providers, are increasing significantly, especially in rural areas (55). March of Dimes found that more than 5·6 million women live in areas with a low access to maternal care or in maternity healthcare deserts, affecting nearly 350,000 births across the United States in 2022 (55). Neonatal care is negatively impacted in these circumstances in which mothers may need to travel long distances immediately postpartum just for a routine check for the infant, which is the first step in establishing medical care and a safety net for the transition to home care.

#### 1.4.4 Neighborhood

The majority of minority groups are clustered in low-income areas with limited health literacy and limited access to healthcare, which eventually contribute to racial and ethnic disparities in medical outcomes (48). Howell et al. noted that Black and Hispanic mothers in New York City tend to give birth in poorer performing hospitals, which can be attributed to access, racial segregation, distance to the hospital, and possibly restricted physician referral (55, 56). Food insecurity, violence, and unstable housing are major barriers not only to the timely access to equitable healthcare but also to the successful implementation of targeted interventions (39, 51, 57). Optimum nutrition for mothers and access to lactation groups/consultants to promote exclusive breastfeeding are among the basic requirements for new mothers along with easy access to vitamin- and iron-fortified formula and pharmacies for basic medications such as paracetamol. Like maternity healthcare deserts, there are pharmacy deserts—defined as being 1 mile from the nearest pharmacy, unless living in a low-income neighborhood where most households do not own a private vehicle, in which case it is a half a mile. These pharmacy deserts are also increasing significantly, especially in low-income and underserved neighborhoods resided in by people of color (58).

#### 1.4.5 Social context

There is a great need to work together as a unit at the community, federal, and national levels to build a community that allows equity in healthcare. Different state policies in maternal healthcare for access by pregnant women feeds to maternal/neonatal transports to higher centers that is subject to financial reimbursements and contributes to the barrier to monitoring, regulation, and standardized care provision (59). One such recent example of policy/law changes was the June 2022 decision to overturn Roe vs. Wade, which maintained under the 14th Amendment that the right to a legal abortion was constitutional (39, 60). Variable interpretations and the adoption of reproductive healthcare policies in different US states once again placed the most vulnerable population at risk. Although a challenge for any pregnant person at many levels, health-literate and financially able people at least have the resources to navigate these challenges relevant to maternal child health.

At the societal level, in addition to acknowledging sex, race(ism), and social determinants in neonatal outcomes, we also aim to raise awareness and highlight possible solutions to improve social health conditions. Although there have been some positive steps toward improving maternal/infant care, a lot more still needs to be done, including taking all the above factors into consideration. The release of the Blueprint for Addressing the Maternal Health Crisis in 2022 activated federal agencies to improve healthcare coverage, increase data collection, and research and encourage the active participation of women (39). The American Rescue Plan Act of 2021 extended postpartum health insurance coverage from 60 days to the entire first year after childbirth (39).

Medical schools in the United States have recognized a drive for curriculums addressing health equity (61). A longitudinal study has shown that the implementation of a curriculum based on direct community involvement significantly increased the confidence of medical students in their knowledge of SDoH and in working with underserved populations (62). Addressing health inequalities in the healthcare system starts with education; providing the appropriate resources from the beginning of a physician's training could be an invaluable tool.

Government agencies and hospitals are acknowledging the importance of SDoH, and 23% of NICUs in the United States have implemented a system built to specifically address SDoH in the main regions based on official recommendations of national organizations as of 2021 (63). Neonatal experts noted that although certain SDoH may be screened for in an NICU setting (e.g., food insecurity and the need for transportation), this may not translate into resources that help alleviate the issue. The experts, in general, advocated for changes in unit and hospital policy that would allow access to additional resources, such as interpreter services, more extensive data collection on online platforms, and the collection of real-time data on patient inequalities, that have the potential to affect statewide and nationwide healthcare policies (64).

Finally, advocating for national policies and funding is paramount in effectively addressing social inequalities in healthcare systems, and the AAP provides a platform on a patient-centered approach through their Screening Technical Assistance and Resource Center. The aim is to provide healthcare workers to improve patients’ health by analyzing various factors, including social determinants of health (65, 66). The importance of social determinants of health and improving patient interactions on a systemic level is a significant issue that should be addressed. However, a lack of resources to systemically implement policies has been cited as a potential reason for the lack of SDoH-specified programs (63, 64). The need to address social determinants of health extends beyond the hospital-wide system, and advocating for resources on a national scale is of the utmost importance.

## 2 Conclusion

In conclusion, we have highlighted the complex interactions between sex, race(ism), and social determinants of health in neonatal outcomes. There is an urgent need for a comprehensive understanding of these factors to improve maternal–neonatal care and reduce disparities in outcomes. Healthcare providers, policymakers, and communities need to work together to combat these complex issues and improve neonatal outcomes for all infants, while understanding the complex interplay between sex, racism, and/or social determinants of health.
